# Adjacent Segment Degeneration after Short-Segment Lateral Lumbar Interbody Fusion (LLIF)

**DOI:** 10.1155/2022/5161503

**Published:** 2022-03-24

**Authors:** Jun Ouchida, Hiroaki Nakashima, Tokumi Kanemura, Yuji Matsubara, Kotaro Satake, Akio Muramoto, Kenyu Ito, Mikito Tsushima, Masayoshi Morozumi, Naoki Segi, Yoshinori Morita, Shiro Imagama

**Affiliations:** ^1^Department of Orthopaedics, Nagoya University Graduate School of Medicine, Nagoya, Japan; ^2^Department of Orthopedic Surgery, Konan Kosei Hospital, Konan, Japan; ^3^Department of Orthopedic Surgery, Kariya Toyota General Hospital, Konan, Japan

## Abstract

**Purpose:**

To investigate the influence on the adjacent segment degeneration (ASD) of short-segment lateral lumbar interbody fusion (LLIF) at 2 years postoperatively.

**Methods:**

Ninety-seven consecutive patients who underwent one- or two-level LLIF were included from two institutions. We diagnosed radiographical adjacent segment degeneration with the appearance of adjacent spondylolisthesis (>3 mm) or deterioration of adjacent disk height (>3 mm) on plain radiographs or decrease of the intervertebral angle (>5 degrees). The differences between the two groups with and without radiographical ASD were investigated using univariate and multivariate analyses to determine the risk factors for ASD. The variables included extent of adjacent decompression, posterior fixation method (open method or percutaneous method), and facet violation on postoperative CT.

**Results:**

In total, 19 patients (19.6%) were diagnosed as radiographical ASD 2 years after surgery. Univariate analysis showed that the ASD (+) group had a high frequency of adjacent decompression (21.1 vs. 3.8%, *p* = 0.035) compared with the ASD (-) group. There were no differences between the two groups in posterior fusion method (percutaneous method 42.1 vs. 57.7%, *p* = 0.221) or facet joint violation (15.8 vs. 14.1%, *p* = 0.860). The multivariate analysis found adjacent intervertebral decompression to be a risk factor for ASD 2 years after surgery (odds ratio: 9.95; 95% confidence interval: 1.2–82.1).

**Conclusions:**

Adjacent intervertebral decompression was considered to be a potential risk factor for the development of ASD after spinal fusion with LLIF.

## 1. Introduction

Lumbar fusion with spinal instrumentation has developed as the gold standard for the surgical treatment of spinal degenerative diseases with instability, deformity, and malalignment, and many clinical studies have reported its favorable results [1–3]. Lumbar fusion surgery carries the risk of degeneration in nonoperated adjacent intervertebral segments due to changes in the kinetic dynamics of lumbar spine mobility, which is a common sequelae called adjacent segment degeneration (ASD) [1, 3–5]. ASD with neurological symptom requires additional surgical treatment that includes extension of the fixation level, which is named as adjacent segment disease. Revision surgery is associated with higher complication rates, higher medical costs and deterioration of patient quality of life, and the etiology of the ASD after lumbar fixation, and the surgical options to prevent them have been discussed in various studies [6, 7]. The etiology of ASD after lumbar fusion is multifactorial, but key factors to prevent ASD have been reported, including adequate postoperative sagittal alignment, intervertebral height restoration, and preservation of posterior stabilizing structures [3, 8–12].

Lateral lumbar interbody fusion (LLIF) for lumbar spinal fusion is a useful technique and is getting widely used for degenerative lumbar diseases. The large footprint of the LLIF cage is a feature of this lateral approach surgery that has been reported to be advantageous for acquisition of local lordosis and restoration of intervertebral height [13]. There is some evidence for indirect decompression with lumbar fixation of short levels using the LLIF cage, which is minimally invasive and provides sufficient postoperative radiographical outcomes in patients with spondylolisthesis [14–16]. However, few reports have investigated the impact on adjacent spinal degeneration after LLIF. Indirect decompression with the LLIF cage, which is characterized by a large footprint, and percutaneous pedicle screws (PPS) enables preservation of the posterior supportive structure affecting the adjacent segment compared with open methods with direct decompression. Therefore, we hypothesized that indirect decompression combined with LLIF and PPS has an advantage in the incidence and severity of ASD. The purpose of this study was to investigate the impact of short-segment LLIF on adjacent segments at 2 years postoperatively. We also investigated the relationship between surgical invasion to the posterior supportive tissues and ASD in indirect decompression using LLIF and PPS by comparing LLIF with direct posterior decompression and PS insertion using the open method.

## 2. Materials and Methods

### 2.1. Subjects and Methods

Ninety-seven consecutive patients who underwent LLIF (mean age 70.1 years, 41 men/56 women) at two surgical institutions between May 2013 and June 2016 were retrospectively included. The objects were cases who underwent short-segment lumbar fixations between 1 and 2 vertebrae levels in spinal degenerative diseases with spondylolisthesis and instability in more than 2 years of follow-up. Patients who underwent 3 or more levels of fixation were excluded. Anterior fixation with an LLIF cage was performed, followed by posterior fixation. With respect to posterior fixation and decompression, there were 2 procedures: (1) the open method (partial laminectomy and pedicle screw fixation were performed with ligament incisions and muscle and soft tissue detachment) and (2) the percutaneous method (pedicle screw fixation was performed percutaneously with PPS without direct decompression at the fixed intervertebral level). The method of posterior spinal fixation and addition or extension of spinal decompression for the adjacent level was determined preoperatively by the surgeon in the patients with spinal canal stenosis on preoperative magnetic resonance imaging.

This study was approved by the Institutional Review Board of Konan Kosei Hospital, Konan, Japan (approved No. 30-045), and written informed consent was waived because of the retrospective design of the study.

### 2.2. Radiographical Evaluation

Postoperative radiographical evaluations for spinal sagittal alignment were conducted using a plane lateral radiograph at 2 weeks after surgery. LL was defined as the angle between the cranial endplates of L1 and the S1 endplate, L4-S angle was defined as the angle between the cranial endplates of L4 and the S1 endplate, and PI-LL mismatch was defined as a difference of more than 10 degrees between PI and LL. The intervertebral height was defined as the average measurement of intervertebral space at the anterior and posterior edges on a plane lateral radiograph. Local lordosis acquisition and local intervertebral height restoration were calculated by the difference in values in the preoperative and postoperative radiographical parameters for each fixed level.

### 2.3. Radiographical Adjacent Segment Degeneration (ASD) and Risk Factor Analysis

On the basis of previous reports, we defined ASD as the proximal adjacent level containing any of the following three conditions [3, 4] on a lateral radiograph: (1) postoperative vertebral slippage of ≥3 mm, (2) narrowing of the intervertebral space of ≥3 mm, or (3) postoperative intervertebral opening of ≥5%. The presence of neurological symptoms at follow-up and reoperation rates at 2 years postoperatively was also investigated.

Differences between the two groups with and without ASD (ASD (+) group and ASD (-) group, respectively) were investigated using univariate and multivariate analyses. The variables used were the patient factors of age and sex, fixed intervertebral number, the methods of posterior fixation (PPS or open), surgical factors such as decompression of the adjacent segment and facet violation of pedicle screw, lumbar lordosis (LL), L4-S angle, pelvic incidence (PI)-LL mismatch, local lordosis acquisition, and local intervertebral height restoration. Facet violation was defined as the presence of screw involvement in the cortical bone of the superior adjacent facet on a postoperative three-dimensional computed tomography (CT) reconstruction view.

### 2.4. Statistical Analysis

All values are expressed as means ± standard deviation. The Mann–Whitney *U* test was used to determine significant differences in age, fixed levels, LL, L4-S angle, local lordosis acquisition, and intervertebral height restoration for the univariate analysis between the two groups. The chi-squared test was used for univariate analysis including sex, posterior fixation methods, adjacent intervertebral decompression, facet joint violation, and PI-LL mismatch. Multivariate logistic regression analysis using the forced input method was performed for variables *p* values less than 0.1 in univariate analyses. Statistical significance was set at *p* < 0.05. The IBM SPSS Statistics version 23.0 software (IBM Corp., Armonk, NY, USA) was used for statistical analyses.

## 3. Results

Among the 97 patients with lumbar spine fusion with LLIF, 44 (45.4%) patients underwent the open method and 53 (54.6%) indirect decompression with PPS. The number of fixed levels was one level in 51 patients and two levels in 46 patients. Adjacent level decompression was performed in 7 patients, all via the open method.  Table 1 shows the characteristics of the patients included in this study.

At follow-up 1 and 2 years postoperatively, 12 (12.4%) and 19 (19.6%) of the patients had ASD, respectively. Two patients had neurological symptoms in a lower extremity that were associated with ASD, and additional decompression surgery was undergone in one patient. In those three cases, none of the direct decompression of the adjacent level was performed at the time of the initial surgery. Overall facet violation was found on postoperative CT in 14 patients (14.4%), which was significantly more frequent in the patients who underwent the open method versus the percutaneous method (25.0% vs 5.7%, *p* = 0.016).

In a comparison of the two groups with and without ASD by univariate analysis, there were no significant differences in age, sex, number of fixed intervertebral vertebrae, posterior fixation, facet violation, postoperative LL, L4-S angle, PI-LL mismatch, local lordosis acquisition, and intervertebral height restoration, whereas the ASD (+) group tended to have more patients with adjacent level decompression (Table 2).

Multivariate analysis, which included adjacent level decompression and LL as variables, showed that only adjacent intervertebral decompression was significantly associated with ASD. The results of the multivariate analysis are shown in Table 3.

## 4. Discussion

The pathogenesis of ASD is multifactorial and that posterior fusion status, injury to the facet joint of the adjacent segment, fusion length, sagittal alignment, age, female sex, and osteoporosis are associated with the progression of postoperative ASD [1]. From a biomechanical standpoint, surgical damage to the posterior stabilizing structures, which include the facet and posterior ligament, has been shown to increase spinal instability and accelerate disc degeneration and progression to ASD [8, 10, 12]. We therefore hypothesized that indirect decompression using an LLIF cage with PPS would avoid injury to the proximal facet joint or posterior structure and contribute to a reduction in ASD progression [17, 18]. However, in the present study, there was no significant association between the two posterior fixation methods of pedicle screws by the open method and PPS or the presence of facet joint violation and the development of radiographical ASD. The results showed that ASD progressed postoperatively in patients with advanced degeneration at the time of surgery who originally had adjacent spinal canal stenosis. We speculate that in patients with advanced degeneration of adjacent intervertebral levels, postoperative degeneration will progress with rigid internal fixation, even if percutaneous method is used to reduce invasion of the posterior structure.

In contrast, the results of the univariate and multivariate analyses showed that extension or concomitant adjacent decompression to proximal of the fusion level was a significant risk factor for ASD. Several previous reports investigating ASD after lumbar fusion have also suggested that extension of adjacent level decompression contributes to the development of disc degeneration and the development of ASD. In a retrospective study of PLIF, Miyagi et al. [9] reported that additional decompression to the adjacent segments of the fusion level was more likely to cause radiological ASD and that patients with ASD had worse clinical outcomes than those without ASD. Ekman et al. [10] reported that laminectomy is a definitive risk factor for disc degeneration of an adjacent level, and Lai et al. [12] noted that damaging the integrity of the posterior complex between the fused segments and the adjacent motion segments leads to the accelerated development of adjacent lumbar instability. The results of the present study of lumbar fusion with LLIF also support these reports. From the results of this study, we discussed that for prevention of early ASD after short-segment lumbar fusion with LLIF, it is necessary to perform imaging studies or diagnostic injection therapy to clarify the responsible level before surgery and avoid unnecessary decompression. In addition, a combination of less invasive methods that can preserve posterior stabilizing structures, such as full endoscopy technique, may be effective in preventing ASD in cases that require the extent of adjacent decompression without fixation [19, 20].

In the present study, 19.6% of the 97 study patients had ASD that were found in the course of a 2-year follow-up after lumbar fusion with a LLIF cage. This was a lower incidence than the 33–82.6% incidence of adjacent intervertebral injury shown in previous studies involving short-segment PLIF at 2 years after the surgery [4, 21, 22]. The importance of lumbar lordosis alignment has been reported in preventing the development of ASD, low LL, and PI-LL mismatch [11, 23]. LLIF has been reported to have several advantages in the formation of adequate lumbar sagittal alignment and in restoring intervertebral height [13]. Although the results of the present study did not show an association between postoperative radiographical parameters, lumbar lordosis, or intervertebral disc height reduction and ASD, we found a the probability of a lower occurrence rate of ASD at 2 years postoperatively for lumbar fixation with LLIF compared to that of PLIF previously reported in terms of ASD. We speculate that even with short intervertebral fixation, the use of a LLIF cage enabled the formation of proper segmental lordosis and may have played a role in reducing the multifactorial development of short-term ASD. Unfortunately, the purpose of this study did not include a comparison of the advantages of the LLIF cage with those of PLIF in terms of lordosis acquisition, and the impact of the use of the LLIF cage on alignment formation is unclear from the present results. Thus, there is a need for comparative studies of PLIF and LLIF matched by surgical technique and patient background.

### 4.1. Limitations

Several potential limitations should be considered in this study. First, this study evaluated only radiographical aspects of ASD, which are insufficient for assessing a causal relationship with patient quality of life and the need for revision surgery. In a study of ASD after PLIF, Nakashima et al. reported that 80% of symptomatic ASDs that resulted in revision surgery occurred 5 years after the primary surgical intervention [3]. This suggests that a follow-up period of 2 years may not be long enough to evaluate mid- to long-term complications after fixation surgery. A study including a longer-term follow-up period will be needed in the future.

Another limitation is the lack of uniformity in the indications for surgery between institutions and surgeons in this study. Because the indications for posterior fixation methods and the extent of adjacent decompression were left to the preoperative surgeon's judgment, potential bias is unavoidable when assessing the impact of these surgical strategies on the study results. Importantly, it is necessary to discuss the possibility that one of the potential biases in the results, i.e., that adjacent decompression was a risk for ASD, was due to the presence of advanced spinal degeneration of an adjacent level, which required surgical intervention preoperatively and that resulted in ASD postoperatively. Ideally, a prospective controlled study is needed to assess the impact of adjacent decompression on ASD. However, we believe that the present results provide sufficient evidence to avoid unnecessary extension of the decompression length and for the need to explain to the patient the possibility of early postoperative ASD, even if the surgeon performs adjacent decompression during lumbar fusion with a LLIF cage.

## 5. Conclusion

Lumbar spinal fusion with LLIF resulted in radiographical ASD in 20% of the patients 2 years after surgery, with adjoining adjacent decompression found to be a risk factor. Although lumbar fusion with the LLIF cage might be useful for the prevention of ASD, studies are needed that include further long-term follow-up of symptomatic complications.

## Figures and Tables

**Table 1:** Patient and surgical characteristics.

N	97
Sex	
Male	41 (42.3%)
Female	56 (57.7%)
Mean age, years	70.3 ± 7.0
>65	77 (79.4%)
Diagnosis	
Degenerative spondylolisthesis	52 (53.6%)
Spinal canal stenosis	42 (43.3%)
Deformity	3 (3.1%)
Type of posterior fixation	
Direct decompression (open PLF)	44 (45.4%)
Indirect decompression (PPS)	53 (54.6%)
Graft materials	
Autografts	44 (45.4%)
Autografts and bone graft substitutes	53 (54.6%)
Number of fixation levels	
1 level	51 (52.5%)
2 levels	46 (47.4%)
Combined with adjacent segment decompression	7 (7.2%)

PLF: posterior lumbar fusion; PPS: percutaneous pedicle screw.

**Table 2:** Results of univariate analysis for developing ASD 2 years after LLIF.

	ASD (+)	ASD (-)	P
N	19	78	
Age, years	70.4 ± 6.6	70.2 ± 7.1	0.901
Sex (female), %	52.6	59	0.616
Number of fixation levels	1.5 ± 0.5	1.5 ± 0.5	0.996
Posterior fixation method (PPS), %	42.1	57.7	0.221
Adjacent level decompression, %	21.1	3.8	0.035
Facet violation, %	15.8	14.1	0.86
Radiographic parameters			
Lumbar lordosis, degrees	38.8 ± 14.9	44.5 ± 12.2	0.091
L4-S angle, degrees	26.0 ± 8.4	25.9 ± 8.6	0.977
PI-LL mismatch, %	52.6	47.4	0.684
△Lordosis, degrees	7.8 ± 7.1	7.1 ± 7.8	0.713
△Intervertebral height, mm	4.6 ± 2.2	5.0 ± 2.8	0.488

ASD: adjacent segment disease at 2 years; PPS: percutaneous pedicle screw; PI: pelvic incidence; LL: lumbar lordosis; △Lordosis: local lordosis acquisition; △Intervertebral height: intervertebral height restoration.

**Table 3:** Multivariate analysis for developing ASD 2 years after LLIF.

	95% CI	OR	P
Adjacent level decompression	1.1-46.9	7.12	0.04
Lumbar lordosis (post op.)	0.93-1.01	0.97	0.16

## Data Availability

The data used to support the findings of this study are available from the corresponding author upon request.
